# Advancing research and clinical care in the management of neuropathic pain after spinal cord injury: Key findings from a Canadian summit

**DOI:** 10.1080/24740527.2017.1385370

**Published:** 2017-12-05

**Authors:** Eldon Loh, Stacey D. Guy, B. Cathy Craven, Sara Guilcher, Keith C. Hayes, Tara Jeji, Phalgun Joshi, Anna Kras-Dupuis, Marie-Thérèse Laramée, Joseph Lee, Swati Mehta, Vanessa K. Noonan, Ethan J. Mings, Michael Salter, Christine Short, Kent Bassett-Spiers, Barry White, Dalton L. Wolfe, Nancy Xia

**Affiliations:** a Parkwood Institute Research, St. Joseph’s Health Care, London, Ontario, Canada; b Lawson Health Research Institute, London, Ontario, Canada; c Toronto Rehabilitation Institute, Department of Medicine, University Health Network, University of Toronto, Toronto, Ontario, Canada; d Rehabilitation Sciences Institute, University of Toronto, Toronto, Ontario, Canada; e Leslie Dan Faculty of Pharmacy, University of Toronto, Toronto, Ontario, Canada; f Centre for Urban Health Solutions, St. Michael’s Hospital, Toronto, Ontario, Canada; g Ontario Neurotrauma Foundation, Toronto, Ontario, Canada; h Rick Hansen Institute, Vancouver, British Columbia, Canada; i Centre Intégré Universitaire de Santé et de Services Sociaux du Centre-Sud-de-l’Île-de-Montréal and Centre de Recherche Interdisciplinaire en Réadaptation, Montréal, Quebec, Canada; j The Centre for Family Medicine, Kitchener, Ontario, Canada; k The Desk Consulting, Oakville, Ontario, Canada; l The Hospital for Sick Children Research Institute, Toronto, Ontario, Canada; m Institute of Medical Sciences, University of Toronto, Toronto, Ontario, Canada; n Faculty of Dentistry, University of Toronto, Toronto, Ontario, Canada; o Department of Physiology, Faculty of Medicine, University of Toronto, Toronto, Ontario, Canada; p Department of Medicine, Division of Physical Medicine and Rehabilitation Dalhousie University, Halifax, Nova Scotia, Canada; q Nova Scotia Rehabilitation Centre, Halifax, Nova Scotia, Canada; r SCI Ontario, Toronto, Ontario, Canada

**Keywords:** health services, neuropathic pain, pain management, research priorities, spinal cord injury

## Abstract

**Background**: Optimal management of neuropathic pain (NP) is essential to enhancing health-related quality of life for individuals living with spinal cord injury (SCI). A key strategic priority for the Ontario Neurotrauma Foundation (ONF) and Rick Hansen Institute (RHI) is optimizing NP management after SCI.

**Aims**: A National Canadian Summit, sponsored by ONF and RHI, was held to develop a strategic plan to improve NP management after SCI.

**Methods**: In a one-day meeting held in Toronto, Ontario, a multidisciplinary panel of 18 Canadian stakeholders utilized a consensus workshop methodology to (1) describe the current state of the field, (2) create a long-term vision, and (3) identify steps for moving into action.

**Results**: A review of the current state of the field identified strengths including rigourously developed evidence syntheses and practice landscape documentation. Identified gaps included limited evidence on NP hindering recommendation development in evidence syntheses, absence of a national strategy, care silos with limited cross-continuum connections, limited consumer involvement, and limited practice standard implementation. The panel identified key themes for a long-term vision to improve the management of SCI NP in Canada, including establishing an integrated collaborative network; standardized care and outcome evaluation; education; advocacy; and directing resources to innovative solutions. The panel identified the next step as prioritization of areas that will have the greatest impact in a 5-year time frame.

**Conclusion**: A strategic plan outlining a long-term vision to improve management of NP after SCI in Canada was developed and will inform future activities of the sponsors.

## RÉSUMÉ

**Contexte**: La gestion optimale de la douleur neuropathique est essentielle à l’amélioration de la qualité de vie liée à la santé des personnes vivant avec une lésion de la moelle épinière. L’une des priorités stratégiques de la Fondation ontarienne de neurotraumatologie et de l’Institut Rick Hansen est l’optimisation de la prise en charge de la douleur neuropathique après une lésion de la moelle épinière.

**Buts**: Un sommet national canadien parrainé par la Fondation ontarienne de neurotraumatologie et l’Institut Rick Hansen a été tenu afin d’élaborer un plan stratégique pour l’amélioration de la gestion de la douleur neuropathique après une lésion de la moelle épinière.

**Méthodes**: Dans le cadre d’une rencontre d’une journée tenue à Toronto, Ontario, un panel multidisciplinaire composé de 18 parties prenantes canadiennes a eu recours à la méthodologie de l’atelier consensuel pour (1) décrire la situation actuelle dans le domaine, (2) définir une vision à long terme et (3) déterminer les étapes à suivre pour passer à l’action.

**Résultats**: Une revue de la situation actuelle dans le domaine a permis d’en répertorier les forces, parmi lesquelles on compte des synthèses de données probantes élaborées avec rigueur et la documentation des pratiques. Les lacunes suivantes ont été répertoriées: des données probantes limitées en ce qui concerne la formulation de recommandations pour empêcher la douleur neuropathique dans les synthèses de données probantes; l’absence de stratégie nationale; un mode de prestation des soins en silos, où les liens entre les continuums sont limités; une participation du consommateur limitée et une mise en œuvre limitée des normes de pratique. Le panel a répertorié les grands thèmes pour élaborer une vision à long terme afin d’améliorer la prise en charge de la douleur neuropathique occasionnée par une lésion de la moelle épinière au Canada, parmi lesquels on compte: l’établissement d’un réseau de collaboration intégré; la normalisation des soins et l’évaluation des résultats; l’éducation; le plaidoyer; et l’attribution de ressources aux solutions novatrices. Le panel a déterminé que la prochaine étape consisterait à prioriser les domaines qui auront le plus de répercussions au cours des cinq prochaines années.

**Conclusion**: Un plan stratégique définissant une vision à long terme pour améliorer la prise en charge de la douleur neuropathique après une lésion de la moelle épinière a été élaboré. Ce plan servira de base aux prochaines activités des promoteurs.

## Introduction

Neuropathic pain (NP) is one of the most common complications after spinal cord injury (SCI)^1–4^ and negatively interferes with rehabilitation, function, mood, and quality of life.^3,5–7^ Though NP is a significant issue for those with SCI, successful clinical management resulting in reduced pain intensity and improved function can be difficult to achieve and implement,^5,6,8^ and evidence for best practices is limited.^9–11^ The recent CanPainSCI Clinical Practice Guidelines (CPG) on the management of NP after SCI,^9–12^ with recommendations on screening and diagnosis, treatment, and models of care, highlight many of these challenges.

The Ontario Neurotrauma Foundation (ONF) and the Rick Hansen Institute (RHI) are Canadian-based nonprofit organizations that seek to improve the quality of life for individuals living with SCI by improving clinical care through research and knowledge translation.^13,14^ On the advice and direction of consumers with SCI, these organizations have identified improvement in NP management as a key strategic priority.^15^ To this end, a one-day summit of key Canadian stakeholders was convened in Toronto, Canada, on November 4, 2016, to advise ONF and RHI on opportunities to improve NP management and to help these organizations develop a strategic plan.

This article reports the results and key themes identified at the summit.

## Methods

### Steering committee

A steering committee (Table 1) was convened in January 2016 and consisted of representatives from the sponsors, a consumer representative, and a research coordinator. The key tasks of the steering committee were to identify a Canadian network of innovation leaders in NP after SCI, decide on the composition of the full summit panel, develop key objectives for the summit, and organize an agenda for the day. The steering committee met monthly via teleconference; a professional facilitator assisted the steering committee in developing the agenda and the objectives for the day.

**Table 1.** Steering committee members of the national SCI neuropathic pain summit.

Committee member	Affiliation	Professional role(s)
Eldon Loh, MD, FRCPC (chair)	Parkwood Institute, London, ON	Physiatrist, pain specialist
Stacey Guy, MSocSc, PhD (candidate)	Parkwood Institute	Research associate
Keith C. Hayes, PhD	Ontario Neurotrauma Foundation	Provincial lead, SCI research, ONF
Tara Jeji, MD	Ontario Neurotrauma Foundation	Program director, ONF consumer representative
Phalgun Joshi, PhD	Rick Hansen Institute	Managing director, RHI
Ethan J. Mings, BMus	Desk Consulting Group, Inc.	Facilitator
Vanessa Noonan, MSc, PhD (PT)	Rick Hansen Institute	Director, research and best practice implementation, RHI
Kent Bassett-Spiers	Ontario Neurotrauma Foundation	Chief executive officer, ONF

SCI = spinal cord injury; ONF = Ontario Neurotrauma Foundation, RHI = Rick Hansen Institute.

An agenda was developed to explore the nature of the issue, identify areas for innovation in the prevention and management of pain across the continuum of care, and develop strategies for implementation of the CanPainSCI CPG across Canada.

### Summit panel

The summit panel consisted of a multidisciplinary group of 18 Canadian individuals with expertise relevant to NP after SCI and included all members of the steering committee (Table 2). Panel members, some with overlapping roles, included physiatrists (three), a family doctor (one), psychologists (two), physiotherapists (two), nursing (one), administrators (five), a knowledge translation researcher (one), a health economist (one), SCI consumer representatives (two), and scientists (three). Panel members from British Columbia, Ontario, Quebec, and Nova Scotia participated.

**Table 2.** Expert panel members of the national SCI neuropathic pain summit.

Panel member	Affiliation	Professional role(s)
Cathy Craven, BA, MD, FRCP(C), MSc, CCD	Toronto Rehabilitation Institute, University Health Network, Department of Medicine University of Toronto, Toronto, ON	Physiatrist, clinician scientist
Sara Guilcher, BSc, MScPT, MSc, PhD	Leslie Dan Faculty of Pharmacy, University of Toronto, Toronto, ON	Physiotherapist, clinician scientist
Anna Kras-Dupuis, RN, MScN, CNN(c), CRN(c)	St. Joseph’s Health Care London, Parkwood Institute, London, ON	Clinical nurse specialist
Marie-Thérèse Laramée, MSc	Institut de réadaptation Gingras-Lindsay-de-Montréal, Montreal, QC	Physiotherapist
Joseph Lee, MD, CCFP, FCFP, MClSc (FM)	McMaster University, Hamilton, ON Centre for Family Medicine FHT, Waterloo, ON	Family physician, Chair (Centre for Family Medicine)
Michael Salter, MD, PhD, FRSC	Hospital for Sick Children, Toronto, ON	Chief of research, scientist
Christine Short, MD, FRCP(C), FACP	Dalhousie University, Halifax, NS	Physiatrist, division head
Barry White	Rick Hansen Institute	Health economist
Dalton Wolfe, PhD	Lawson Health Research Institute, Parkwood Institute, London, ON	Scientist
Nancy Xia, BSc	SCI-Ontario	Consumer representative

SCI = spinal cord injury.

### Process

Prior to the meeting, the summit panel reviewed and agreed to the agenda and objectives established by the steering committee. Panelists were asked to provide their position, institution, degrees, and any potential external conflicts to the chair. If any potential conflicts of interest were identified by the chair during the meeting, panelists could be excused from that portion of the discussion. Panelists were free to remove themselves from portions of the discussion if it was felt that they had a conflict.

On the day of the meeting, introductory comments were given by the chair, sponsors, and a consumer representative to provide appropriate background and context for the specific goals of the session. A consumer representative provided a detailed statement on the lived experience of NP after SCI.

The specific goals of the summit were to:

1. Describe the current state of the field: Identify the panel’s experiences and knowledge about NP in the domains of research, clinical practice/implementation, and the consumer experience. From this description, list what we are doing well in the field and any gaps that may exist in the above domains.
2. Create a long-term vision: Develop a shared practical vision of results to be achieved in NP after SCI.
3. Move into action: Identify key challenges that should be taken into consideration when building a plan to achieve the vision and suggested next steps to continue the work.

The Technology of Participation Consensus Workshop methodology,^16^ which involves small group breakout sessions followed by a facilitated large group discussion, was utilized to generate specific items and general thematic areas related to the goals above. These items and theme areas were later compiled and documented.

### Sponsors and organizations

The summit was sponsored and funded by ONF and RHI. SCI-Ontario, a consumer advocacy group for those living with SCI, was also represented on the summit panel.

## Results

The Summit was held November 4, 2016, at the ONF offices in Toronto, Canada. A consumer representative’s (TJ) opening remarks provided an account of her experience with pain after SCI. She emphasized that “… pain is part of me and many of my peers … for some it’s experienced as worse than the injury itself.” She related that alternatives to medications are important to her and other individuals with SCI and that because of side effects and limited efficacy with pharmacological treatments, “they spend time and money searching for additional treatments.” This account informed and grounded the discussion for the day.

Each summit objective was discussed in detail, and themes within each area were generated. The key points addressed are presented here.

### Current state of the field

The panel considered their own experiences to generate a list of current activities within the field of NP after SCI. Many of the specific items identified applied to multiple domains.

Thematic areas related to the current state of the field included the following:

- Evidence syntheses
- Practice landscape
- Clinical resources
- Consumer engagement

#### Evidence syntheses

Within the research domain, two key thematic areas identified were evidence syntheses and practice landscape studies (see below). Evidence syntheses were recognized as a particular strength of SCI research within Canada. High-quality evidence syntheses that were identified included the CanPainSCI CPG,^9–12^ the Spinal Cord Injury Research Evidence (SCIRE) website,^17^ and the *Canadian Guideline for Safe and Effective Opioid Use for Chronic Non-cancer Pain*.^18^ The CanPainSCI CPGs^9–12^ were recently developed in accordance with the AGREE II protocol and provide recommendations on screening and diagnosis, treatment, and models of care. International research collaborations, including those involving the CanPainSCI CPG, were noted to be a strength.

SCIRE is a website that provides systematic reviews on the available evidence in SCI rehabilitation in different topic areas, including pain. The Canadian opioid guideline^18^ was not specifically developed for the SCI population but provides useful recommendations for opioid prescription in general.

#### Practice landscape

Two studies documenting the practice landscape across Canada were noted: the Healthcare Provider Survey^19^ and the Rehabilitation E-scan.^20^ The Healthcare Provider Survey^19^ was completed in 2014 and solicited the opinions of various clinicians on the nature of pain following SCI, as well as the resources used to guide management of pain following SCI. The Rehabilitation E-scan^20^ obtained data on rehabilitation services and practices across 12 sites in Canada relating to secondary complications following SCI, including neuropathic pain. In the E-scan, it was found that standardized assessments for diagnosis and treatment and standardized treatment guidelines were used infrequently.^20^ Only five out of 12 sites routinely used standardized assessments for diagnosis or treatment, and only three out of 12 sites followed a standard of care.^20^

Given the lack of standardization in the provision of care for NP across Canada, the SCI Knowledge Mobilization Network^21^ seeks to implement standardized practice in pain assessment at six rehabilitation sites across Canada. The implementation of internationally accepted standards of care, such as the SCI Pain Basic Dataset,^22^ is among its goals.

Efforts to document the characteristics of those undergoing acute rehabilitation and community care of NP after SCI across Canada are ongoing, such as the Rick Hansen Spinal Cord Injury Registry.^23^ This registry collects demographic data and the characteristics of an individual’s pain after SCI over time, using elements of the SCI Pain Basic Dataset, to improve clinical care and outcomes.^23^

#### Web-based and in-person clinical resources

Both web-based and in-person clinical resources were identified to help clinicians and those with SCI manage pain. Web-based resources identified include the SCI Pain Navigator^24^ and SCI-U.^25^ The SCI Pain Navigator^24^ is an online decision support tool developed in Australia that assists clinicians in managing pain after SCI. SCI-U^25^ is a series of online courses for SCI consumers that provides information on medical issues after SCI, including pain.

In-person resources identified were primarily educational resources for patients, such as the Living Engaged and Actively With Pain^26^ program at the University Health Network (Toronto, Canada) and peer support/mentorship services.

#### Consumer engagement

Partnerships with consumer organizations, such as SCI Canada and its provincial affiliates, in project development and organizational planning were recognized as a particular strength.

#### What are the gaps?

Though numerous strengths relating to SCI NP management were documented, the panel identified significant gaps that need to be addressed in order to advance clinical care.

The gaps identified were grouped into five thematic areas:

1. Limited evidence and research.
2. Absence of a national strategy.
3. Limited connections across the care continuum.
4. Limited consumer involvement.
5. Practice standard integration, including the CanPainSCI CPGs.

#### Limited evidence and research

Though the generation of evidence syntheses was identified as a strength, the panel acknowledged that the limited evidence and research forming the basis of these reviews can hinder the development of effective recommendations and guidelines. As a result, panel members perceived a chasm between the available evidence, including guideline recommendations, and current clinical practice. A lack of evidence was noted in pharmacological and nonpharmacological treatment options, as well as in biomarkers to diagnose NP and track treatment response. The CanPainSCI CPG also highlighted this lack of evidence in SCI NP.^9–12^ The panel identified a lack of funding, particularly for nonpharmacological management options, as a barrier to new evidence generation. Limitations in the current literature regarding the economic consequences of NP in SCI populations was also noted.

The panel identified research into NP after nontraumatic SCI as important. Much of the focus of NP research has been on the posttraumatic population, but the relative proportion of individuals admitted to tertiary Canadian SCI rehabilitation units with nontraumatic SCI has been increasing.^27^

Despite the limited evidence available, there is no established formal interaction between the diverse research fields needed to address the issue of SCI NP in Canada. Therefore, there is an insufficient Canadian “research pipeline” to address knowledge deficiencies in SCI NP.

#### Absence of a national strategy

The lack of a visible national strategy, including a national implementation strategy, makes it difficult to standardize practice nationally, with care delivery varying between different provinces/territories and between urban and rural locations. Additionally, the absence of a national strategy makes collaboration between stakeholders such as researchers, clinicians, and consumers more difficult. The collaboration between the Alberta Bone and Joint Institute,^28^ the McCaig Institute for Bone and Joint Health,^29^ and the Bone and Joint Health Strategic Network^30^ is an example of a network that exists to move research into the clinical realm and may be a useful model to emulate.

#### Limited connections across the care continuum

The panel noted the lack of networking and communication between providers across the care continuum as a rate-limiting step in implementing comprehensive NP care for those with SCI. There are a variety of synergies and collaborations that have not been utilized and would improve care for those with NP, such as Project ECHO^31^ and e-consults.^32^ Streamlining NP treatment plans and goals across the continuum from acute care to rehabilitation to the community was also recognized as an important goal.

Collaboration with specialized pain clinics in the provision of clinical care was emphasized, and the panel felt that it was important to develop synergies with these clinics. Specialized pain clinics have the infrastructure to provide nonpharmacological pain treatments and multidisciplinary treatment protocols that may not be available in tertiary SCI rehabilitation centers but may lack specialization to manage issues specific to SCI.

The panel noted that equitable access to specialized care for assessment and management of NP after SCI can be difficult, particularly for those who live in a rural setting.^33^ In terms of access to specialized pain clinics, waiting times can be prolonged, and regional availability of these clinics differs.^34^ Furthermore, SCI rehabilitation hospitals within Canada are primarily situated in larger urban areas, and long-term follow-up for those with SCI can be challenging in general.^33^

#### Limited consumer involvement

The disadvantage of limited SCI consumer involvement in ongoing research, implementation, and visioning projects, among other activities, is that the priorities of SCI consumers may not be addressed. Efforts to improve and maintain consumer engagement were identified as an important goal moving forward.

Within the SCI community, there has been limited advocacy for those with NP despite a widespread prevalence.^1,3,4,12^ To improve resource allocation and dedicated research within the field, promoting awareness and improved advocacy of NP after SCI was emphasized by the panel.

#### Practice standard implementation

The CanPainSCI CPGs were recently published and provide recommendations for NP after SCI. As the CPG process moves toward the implementation phase, several challenges were identified. Barriers such as local hospital policies, diversity in provincial drug formularies, and clinician engagement were discussed by the summit panel. Funding resources and personnel to guide the implementation were recognized as a necessity.

Partnerships with other organizations involved in their own implementation processes will also be important to disseminate practice standards specific to NP management after SCI.

### Long-term vision

Based on the strengths and gaps in SCI NP management, the summit panel discussed the elements of a long-term vision that should guide ONF and RHI over the next 5 years. The key thematic areas of this long-term vision are as follows:

- Establishing an integrated collaborative network
- Standardization through implementation
- Standardized evaluation of impact
- Empowerment through education
- Advocacy
- Directing resources to realize innovative solutions

#### Establishing an integrated collaborative network

The infrastructure for a Canadian SCI network (including Rick Hansen Spinal Cord Injury Registry, SCIRE, and Knowledge Mobilization Network) exists to integrate research and care and may provide an opportunity to advance NP management in Canada. Integration of existing resources will be important to generate a cross-country network of consumers, clinicians, and researchers focused on improving NP management after SCI.

Collaborations with external organizations, such as the MS Society, Canadian Pain Society, and Canadian Institutes of Health Research Strategy for Patient-Oriented Research network were proposed as ways to diversify and strengthen the network. Incorporating the work of those examining NP, but not necessarily in those with SCI, was felt to be important in bringing a new perspective to ongoing efforts in enhancing care.

In conjunction with a collaborative network, developing a pilot project of one or two interdisciplinary centers of excellence was suggested as a means to clinically integrate the advice and recommendations of the network.

#### Standardization through implementation

Implementing a standard of care nationally for those with NP after SCI should include implementation of common assessment tools and outcome measures, as well as treatment care pathways. The involvement of organizations such as Accreditation Canada^35^ would aid future implementation efforts.

Implementation of guideline recommendations would be an important step in initiating the standardization process. A “Living Guidelines” pilot project, which is currently being undertaken as a collaboration between Canadian and Australian research groups, seeks to frequently (every 6 months) reevaluate and update guideline recommendations related to NP after SCI. The incorporation of this type of “living document” could add significant value to optimal standardized care nationally.

#### Standardized evaluation of impact

Application of standards for the clinical and economic evaluation of SCI NP management was recognized as vitally important by the summit panel. The panel members emphasized the importance of selecting appropriate analytic methods and outcome measures for the evaluation of preventative and therapeutic solutions to the burden of NP. More rigorous and comparable evidence in this regard is expected to lead to more defensible decisions involving the allocation of resources for SCI NP management and research.

The panel recommended a greater focus on assessment of the degree to which preventative and therapeutic options effect (or are expected to effect) the burden of NP after SCI, as defined by quantifiable patient-reported outcome measures, excess expenditures, or use of health care resources associated with SCI NP.

#### Empowerment through education

The summit panel advocated for improved knowledge of NP management after SCI among clinicians to optimize provision of care and among consumers to optimize self-management. For clinicians, strategies to improve awareness and knowledge might include educational modules for primary care providers, enhanced postgraduate curricula to include NP after SCI, and dissemination of the CanPainSCI CPGs in a format that facilitates implementation (i.e., evidence-informed protocols). For consumers, plain-language medication guides, plain-language versions of the CanPainSCI CPGs, and self-management modules were suggested.

#### Advocacy

Advocacy at numerous levels for SCI NP management was emphasized by the summit panel. Policies at the national level to ensure equitable access to pain services and expertise were suggested, including improved access and funding for pharmacological and nonpharmacological therapies (acknowledging current variations in drug coverage and provincial formularies as a significant source of practice inequity).

#### Directing resources to realize innovative solutions

Building capacity to enhance the pipeline of next practices was emphasized by the summit panel. Capacity should be built across the continuum, from basic science to policy development. An environmental scan of current research projects and allocated funding would be a useful first step. Additionally, building partnerships with industry to evaluate novel treatments was suggested by the panel members.

### Moving into action

Numerous items and themes were generated by the visioning exercise. The challenge moving forward will be to prioritize those areas that will have the greatest impact in a reasonable time frame with a predictable economic burden. Criteria for prioritization should include clinical impact, alignment with provincial agendas, alignment with consumer expectations and priorities, and capacity for implementation.

An SCI NP working group will be convened to identify priorities that should be pursued from the themes and items generated by the summit panel and to map out funding opportunities. The working group should reflect diverse disciplines, and consumer involvement is a necessity.

## Discussion

This article presents the findings and recommendations of a one-day national Canadian summit on NP management after SCI. Based on the current strengths and gaps in the field, the summit panel generated six thematic areas representing a practical, long-term vision of results that ONF and RHI should strive to achieve in NP after SCI. The summit panel agreed that adherence to these themes will help to improve the management of NP after SCI in Canada. Key priorities within each theme should be easily implemented, have significant clinical impact, and align with consumer priorities.

The national summit followed a consensus-based methodology, involving key stakeholders from across Canada, and necessarily reflected the perspectives and experiences of the panel members. Though it was not possible at this time to include all relevant disciplines at the summit (e.g., industry, acute care, neurosurgery, social work, pharmacy), their involvement moving forward will be solicited.

Although the focus of the summit was on NP after SCI in Canada, many of the panel’s findings are relevant to the SCI community internationally, such as the limited evidence base for NP treatment. In addition, many of the themes generated by the summit panel may apply to other chronic pain conditions^36,37^ and chronic conditions in general.^38^ Other organizations generating long-term research and clinical goals for management of chronic pain and other chronic conditions may find the thematic areas generated by this summit useful.

The SCI NP summit represents only one of four national summits in SCI commissioned by ONF and RHI, with additional summits in family practice, pressure ulcers, and bladder health. The leads from each summit will create a larger strategic plan for advancing SCI care in Canada. This is particularly important because secondary complications following SCI are not isolated issues and must be evaluated in the context of other comorbidities—for example, pain may be exacerbated by an underlying bladder issue or pressure ulcer.

In conclusion, the panel recommended a long-term vision focused on principles of increased collaboration between stakeholders, standardization of care, standardized evaluation of outcomes, advocacy, education, and knowledge generation to improve the management of NP after SCI. The next challenge will be prioritizing and implementing specific items within each thematic area to maximize impact for those with SCI. As emphasized by a consumer representative on the panel, NP can be a significant and challenging issue for individuals with SCI, and the perspective of the consumer should be central to guiding future work in the area.
